# Post‐pollination barriers contribute to coexistence of partially pollinator‐sharing *Arisaema* species (Araceae)

**DOI:** 10.1002/ece3.10696

**Published:** 2023-11-02

**Authors:** Luo Zeng, Wei‐Jie Shu, Hua He, Tao Li, Xiao‐Chen Yang, Li Li

**Affiliations:** ^1^ College of Biology and Environmental Sciences, Jishou University Jishou Hunan China

**Keywords:** *Arisaema*, fungus gnat, pre‐ and post‐pollination barriers, reproductive isolation, species coexistence

## Abstract

Reproductive isolation plays an important role in maintaining the species integrity of sympatric close relatives. For sympatric *Arisaema* species, interspecific gene flow is expected to be effectively prevented by pre‐pollination barriers, particularly strong pollinator isolation mediated by fungus gnats. However, due to the lack of quantitative studies combining multiple pre‐ and post‐pollination barriers, it is not known whether pre‐pollination isolation is complete, and whether post‐pollination barriers also contribute to reproductive isolation among some *Arisaema* species. In this study, we quantified the individual strengths and absolute contributions of four pre‐ and post‐pollination barriers (phenological isolation, pollinator isolation, hybrid fruit formation, and hybrid seed formation) among three sympatric *Arisaema* species (*A. bockii*, *A. lobatum*, and *A. erubescens*). Although phenological isolation and pollinator isolation reduced the frequencies of interspecific pollen transfer among these species, the partial overlap of flowering times and pollinator assemblages resulted in incomplete pre‐pollination isolation. Post‐pollination barriers also contributed to reproductive isolation at the hybrid fruit and seed formation stages. We propose that, although pre‐pollination barriers are expected to contribute more to total isolation than post‐pollination barriers in *Arisaema*, pre‐pollination barriers may not completely prevent interspecific pollen transfer among some *Arisaema* species. Post‐pollination barriers, which are generally ignored, may also have contributed significantly to reproductive isolation in *Arisaema*.

## Graphical abstract

We quantified multiple pre‐ and post‐pollination barriers and their absolute contribution to total reproductive isolation among three sympatric *Arisaema* species (*A. bockii*, *A. lobatum*, and *A. erubescens*). Our results revealed pre‐pollination isolation that does not adequately prevent interspecific hybridization among these species. Post‐pollination barriers also contributed to reproductive isolation. Our study also highlights the importance of a combination of pre‐ and post‐pollination barriers for species coexistence in *Arisaema*.

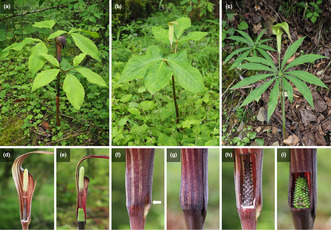

## 1 INTRODUCTION

Mechanisms of reproductive isolation are crucial in maintaining the species integrity of sympatric close relatives by reducing interspecific gene flow (Christie & Strauss, 2019; Gilman & Behm, 2011; Rieseberg et al., 2006; Todesco et al., 2016; Weber & Strauss, 2016). In plants, mechanisms promoting reproductive isolation can be classified into pre‐ and post‐pollination barriers (Baack et al., 2015). Pre‐pollination barriers are associated with adaptive ecological divergence, including ecogeographic, phenological, and pollinator isolation; post‐pollination barriers are associated with the accumulation of genetic incompatibilities, including gametic incompatibilities, conspecific pollen precedence, hybrid inviability, hybrid sterility and hybrid breakdown (Baack et al., 2015; Rieseberg & Willis, 2007). The reproductive barriers function sequentially in life history, and early‐acting barriers can weaken the contribution of late‐acting barriers to total isolation (Coyne & Orr, 1989). Pre‐pollination barriers generally exhibit greater strength and make a more significant contribution to total isolation when compared to post‐pollination barriers, as evidenced by the analyses and generalizations of empirical data (Christie et al., 2022; Lowry et al., 2008). For example, in most orchids, pre‐pollination barriers have been found to be strong while post‐pollination barriers often contribute less to total isolation (Baack et al., 2015; Sun et al., 2015; Whitehead & Peakall, 2014). However, in some cases post‐pollination barriers were stronger and contributed more to total isolation than pre‐pollination barriers: Strong post‐pollination pre‐zygotic barriers acting at stigmatic level guaranteed complete reproductive isolation between *Orchis italica* and *O. papilionacea*, while pre‐pollination barriers were very weak or absent (Pellegrino et al., 2010). The pattern of reproductive isolation varies among species pairs. Therefore, it is important to quantify multiple pre‐ and post‐pollination barriers and their contribution to total isolation for understanding the coexistence of sympatric closely related species (Sobel & Chen, 2014).

*Arisaema* Martius is a large genus in Araceae, containing around 200 deciduous or evergreen perennial herbs (Ohi‐Toma et al., 2016). Most species are distributed in subtropical to cool temperate regions of Asia and several species are endemic to North America and tropical East Africa (Gusman & Gusman, 2006; Murata, 2011). In many regions, *Arisaema* species often overlap in their distribution (Matsumoto et al., 2018; Murata, 1995; Serizawa, 1988, 1997). It is generally accepted that interspecific gene flow among sympatric *Arisaema* species is effectively prevented by pre‐pollination isolation (Matsumoto et al., 2019, 2021; Murata et al., 2018). Divergence of habitat and flowering time reduced interspecific gene flow to some extent among sympatric *Arisaema* species (Matsumoto et al., 2019, 2021; Murata & Ohno, 1989). In contrast to relatively weak ecogeographic isolation and phenological isolation, selective fungus gnat visitation resulted in strong pollinator isolation among some *Arisaema* species (Kakishima et al., 2019, 2020; Matsumoto et al., 2019, 2021; Suetsugu et al., 2021). The strong pollinator isolation among certain *Arisaema* species might serve as a compensatory mechanism for weakened early‐acting barriers, thereby ensuring the prevention of interspecific hybridization (Matsumoto et al., 2021).

However, some natural hybridization occurred (Hayakawa et al., 2011, 2013; Kobayashi et al., 2005; Lee et al., 2011; Maki & Murata, 2001; Murata & Ohno, 1989; Sanders & Burk, 1992), suggesting that pre‐pollination isolation was incomplete among some *Arisaema* species. In artificial crossing experiments among various Japanese *Arisaema* species, F1 hybrids had high germination rates and high pollen fertility (Murata et al., 2018; Murata & Ohno, 1989). Thus, subsequent studies treated post‐pollination isolation as weak or absent among these species (Matsumoto et al., 2019; Suetsugu, 2022; Suetsugu et al., 2021). However, since the mechanism of post‐pollination isolation at other stages has not been studied, we do not know whether there are strong post‐pollination barriers among *Arisaema* species. The mechanisms of coexistence among sympatric *Arisaema* species are not well understood due to the lack of quantitative studies combining multiple pre‐ and post‐pollination barriers.

In this study, we focused on three sympatric congeners in the genus *Arisaema*: *A. bockii*, *A. lobatum*, and *A. erubescens*. We used a combination of approaches including field observations and experiments to clarify the mechanism of reducing interspecific gene flow, and then to quantify the strength of multiple pre‐ and post‐pollination barriers and their contribution to total isolation. In doing so, we hope to solve the following questions: (1) what are the strengths of different isolation barriers? (2) What is the contribution of pre‐ versus post‐pollination barriers to the maintenance of species integrity? Our assessment of reproductive isolation barriers among these sympatric species will provide new insights toward a better mechanistic understanding of sympatric coexistence of *Arisaema* species.

## 2 MATERIALS AND METHODS

### 2.1 Plant materials

**FIGURE 1:**
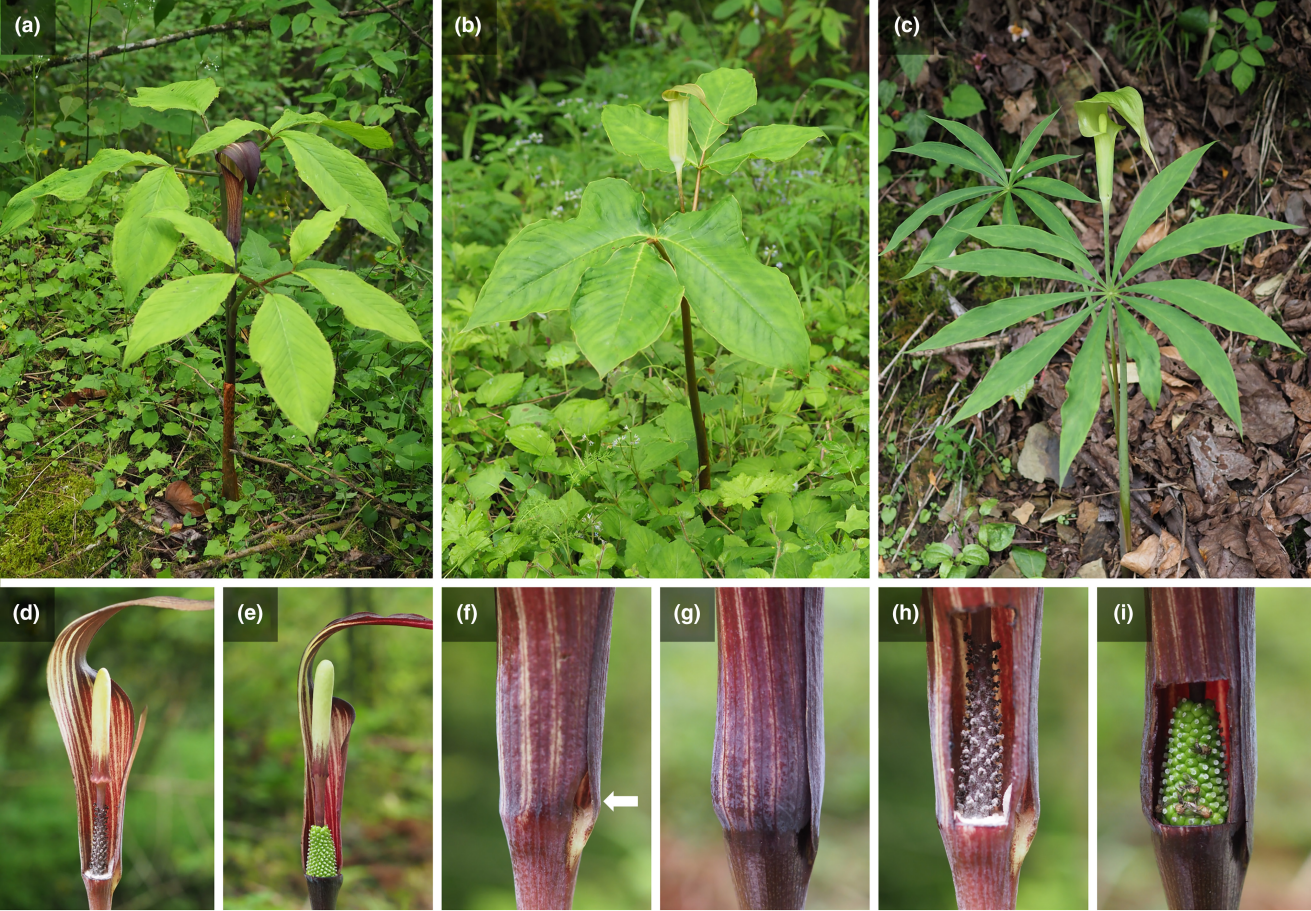
Three examined *Arisaema* species in the Badagongshan Reserve. (a) *A. bockii*. (b) *A. lobatum*. (c) *A. erubescens*. (d–i) Similar inflorescence structure and pollination strategy of *Arisaema* species, using *A. bockii* as an example. (d) and (e) Vertical section of male and female inflorescence, respectively. (f) An exit hole in bottom of the male spathe. (g) The closed bottom of the female spathe. (h) Male spadix. (i) Female spadix with dead fungus gnats.

In this study, we focused on three dioecious *Arisaema* species: *A. bockii* and *A. lobatum* from the section *Pistillata*, and *A. erubescens* from the section *Sinarisaema*. All species are geophytes, with the shoots reproducing during the growing season and then dying, leaving the underground rhizomes dormant through the winter. The chromosome number of *A. bockii* is 2n = 26 (Li et al., 2010), while the chromosome numbers of *A. lobatum* and *A. erubescens* are 2n = 28 (Petersen, 1989; Watanabe et al., 2008). Although the shape and color of inflorescence are significantly different among the three species (Figure 1a–c), the structure of inflorescence is similar. The inflorescence consists of a spadix with fertile flowers at the base and a well‐developed sterile appendix above, and a spathe that wraps around the spadix and forms a hood covering the spadix tip (Figure 1d,e).

Dioecious *Arisaema* species possess a pitcher‐trap pollination system (Barnes, 1935; Vogel & Martens, 2000). In this pollination system, the odor emitted from the spadix appendage and/or the spathe attracts fungus gnats (Mycetophilidae and Sciaridae) into the tubular spathe (Barnes, 1935; Kakishima et al., 2019; Suetsugu et al., 2021, 2022; Vogel & Martens, 2000). Attracted fungus gnats slip down to the bottom of the spathe because the spadix appendage and inner surface of the spathe are covered with wax (Vogel & Martens, 2000). The male spathe has an exit hole at the bottom, through which the captured fungus gnats can escape with pollen grains (Figure 1f,h). However, the female spathe has no exit hole, and the captured fungus gnats move around the female spadix, deposit pollen grains on the stigma, and then die (Vogel & Martens, 2000) (Figure 1g,i). This pollination strategy was also employed in all three species studied, where dead fungus gnats were found in the female spathes without exit holes, but not in the male spathes with exit holes (Figure 1h,i).

### 2.2 Study site

The study was conducted at Badagongshan National Nature Reserve, Hunan Province, China (29°47′06″ N, 110°05′33″ E; 1369 m a.s.l.). At the study site, sympatric populations of the three *Arisaema* species commonly occurred in forest margins. There was no discernible difference in microhabitat among the three species, and heterospecific individuals often co‐occurred within a distance of less than 5 m. In the survey, we found three putative hybrids with an intermediate morphology between *A. bockii* and *A. lobatum* (Figure S1).

### 2.3 Phenological isolation

To assess the differences in flowering time among our three *Arisaema* species, their flowering phenology was recorded in a mixed community of the three species. Sixty *A. bockii* (25 female and 35 male), 64 *A. lobatum* (18 female and 46 male), and 79 *A. erubescens* (21 female and 58 male) were observed every day from April 10, 2022, to May 29, 2022 (Table S1). The flowering period began when the spathe opened above and ended when it faded and/or withered. Fewer females were observed than males because the sex ratio of *Arisaema* populations is usually biased toward males (Richardson & Clay, 2001).

### 2.4 Pollinator isolation

To investigate the differences in floral visitor assemblage among the three *Arisaema* species, floral visitors of these species were collected daily from the inflorescences of each individual used in the investigation of flowering phenology from the beginning to the end of the flowering period using a hand‐made aspirator. The exit holes of the male spathes were plugged with cotton before the male spathes opened to prevent the escape of the captured floral visitors. In 2022, we collected 588 arthropods from *A. bockii*, 315 from *A. lobatum*, and 697 from *A. erubescens* (Table S2). The collected arthropods were fixed in a freezer overnight and dried at room temperature (~10–30°C). The samples were observed under a stereomicroscope (M205C; Leica Microsystems, Cambridge, UK) and identified to family for dipterans and to genus for fungus gnats, using identification manuals (McAlpine et al., 1981, 1987), Other arthropods were identified to order rank only.

### 2.5 Post‐pollination barriers: Hybrid fruit and seed formation

To test for the effect of pollen source (intra‐ vs. interspecific) on fruit and seed formation, interspecific and intraspecific crosses were conducted in the mixed community of three *Arisaema* species in 2022. For each *Arisaema* species, 30 female inflorescences were bagged. With the spathes opening above, half of the inflorescences (15) were brushed with conspecific versus congeneric #1 pollen, while the other half (15) were brushed with conspecific versus congeneric #2 pollen. Approximately 3 months after pollination, more than 150 fruits were collected for each cross. Large and plump fruits were recorded as setting fruits, while small and wrinkled fruits were not. Large and aborted embryos were counted per setting fruit under a stereomicroscope. The rates of setting fruits and large embryos were calculated to assess fruit and seed formation (Table S3).

### 2.6 Quantifying reproductive isolation

To determine the strength of individual barriers and their contribution to total isolation, reproductive isolation was quantified by a unified approach directly related to gene flow proposed by Sobel and Chen (2014).

The strength of phenological isolation was calculated following equation 4A of Sobel and Chen (2014):

(1) $${\mathrm{RI}}_{4A}=1-2\times \left(\frac{H}{H+C}\right)$$

where H and C represent the mean number of flowering days in which the female plant of each *Arisaema* species overlapped with the heterospecific and conspecific male plant, respectively. $\frac{H}{H+C}$ represents the probability of heterospecific gene flow, *P*(GF).

The strength of pollinator isolation was calculated with reference to equation RI_4A_ in Sobel and Chen (2014) as follows:

(2) $$\begin{array}{c} \mathrm{RI}=1-2\sum_{i}\left(\frac{C_{Fi}}{C_{F}}\times \frac{H_{Mi}}{H_{Mi}+C_{Mi}}\right) \end{array}$$

where $C_{Fi}$ and $C_{F}$ represent the mean number of floral visitors for taxa *i* per day captured by the female plant of each *Arisaema* species and the mean number of all flower visitors per day captured by the female plant of each *Arisaema* species, respectively. $H_{Mi}$ and $\;C_{Mi}$ represent the mean number of flower visitors for taxa *i* captured per day by the heterospecific and conspecific male plant, respectively. Arthropods taxon that could not be identified were not included in the calculations. $\sum_{i}\left(\frac{C_{Fi}}{C_{F}}\times \frac{H_{Mi}}{H_{Mi}+C_{Mi}}\right)$ represents the probability of heterospecific gene flow due to floral visitors for all taxa, *P*(GF).

The strength of the isolation barriers to hybrid fruit and seed formation were calculated following Equation 1, For the isolation barrier to hybrid fruit formation, *H* and *C* represent the fruit sets of the interspecific and intraspecific crosses, respectively. For the isolation barrier to hybrid seed formation, *H* and *C* represent the seed sets of the interspecific and intraspecific crosses, respectively.

The cumulative strength of multiple sympatric barriers was calculated based on equation RI_4S3_ in Sobel and Chen (2014) as follows:

(3) $$\begin{array}{c} {\mathrm{RI}}_{4S3}=1-2\times \left(\frac{\prod_{i=1}^{n}P{\left(H\right)}_{i}}{\prod_{i=1}^{n}P{\left(H\right)}_{i}+\prod_{i=1}^{n}P{\left(C\right)}_{i}}\right) \end{array}$$

where *n* is the number of barriers under consideration, and $P{\left(H\right)}_{i}$ and $P{\left(C\right)}_{i}$ are the probabilities of heterospecific and conspecific gene flow respectively for each barrier, *i*.

The absolute contribution of each individual barrier to total isolation were calculated by equation ${\mathrm{AC}}_{i}$ from Sobel and Chen (2014) as follows:

(4) $$\begin{array}{c} {\mathrm{AC}}_{i}={\mathrm{RI}}_{\left[1,i\right]}-{\mathrm{RI}}_{\left[1,i-1\right]} \end{array}$$

where ${\mathrm{RI}}_{\left[1,i\right]}$ denotes the combined isolation calculated by ${\mathrm{RI}}_{4S3}$ including all barriers from the first to act (1) through the focal barrier (*i*), and ${\mathrm{RI}}_{\left[1,i-1\right]}$ denotes the same calculation omitting the focal barrier.

### 2.7 Statistical analysis

All statistical analyses were performed with R (version 4.3.1.; R Core Team, 2021). Interspecific differences in flowering time were tested using the Kruskal–Wallis test followed by a posteriori Dunn's test with the package “rstatix” and “dunn. test,” because the dataset did not follow a Gaussian distribution (Shapiro–Wilk test, all *p* < .05). The interspecific differences in the floral visitor assemblage were examined by permutational multivariate analysis of variance (PERMANOVA) (Bray–Curtis dissimilarity index, 999 permutations) with the package “vegan.” When the floral visitor assemblages were significantly different among species, we conducted pairwise PERMANOVA with Bonferroni correction for all species pairs. The effect of pollen source (intra‐ vs. interspecific) on fruit set and seed set were tested by GLMs (binomial distribution and logit link function) with the package “arm,” because the datasets for fruit set and seed set align with the definition of a binomial distribution.

## 3 RESULTS

### 3.1 Phenological isolation

**FIGURE 2:**
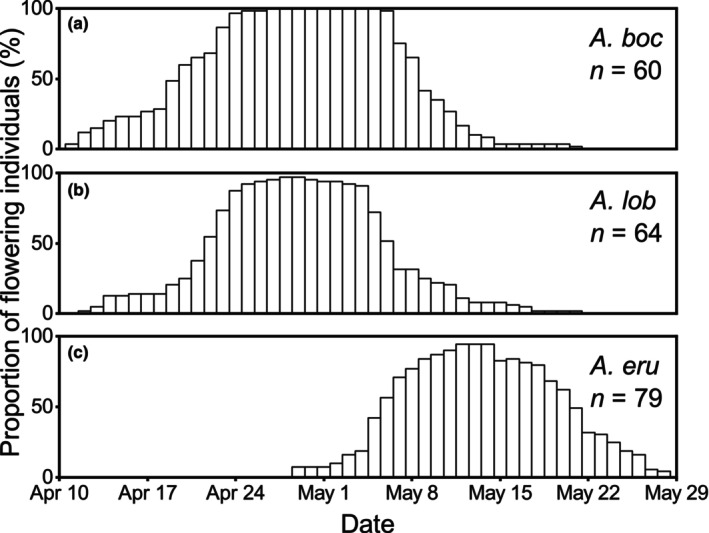
Flowering phenology of *Arisaema bockii* (a), *A. lobatum* (b) and *A. erubescens* (c) in the Badagongshan Reserve. Species abbreviations: *boc*, *bockii*; *lob*, *lobatum*; *eru*, *erubescens*.

The dates of initial flowering differed significantly among the three *Arisaema* species in the Badagongshan Reserve (*p* < .001). The mean date of initial flowering of *A. bockii* (day of year: 109.27 ± 4.16) was earlier than that of *A. lobatum* (111.50 ± 3.80) by 2 days with no significant differences (*p* = .06), and they were significantly earlier than that of *A. erubescens* (127.37 ± 4.32) by 18 and 16 days, respectively (both *p* < .001, Figure S2). The end dates of flowering also differed significantly among the three *Arisaema* species (*p* < .001). The end date of flowering of *A. bockii* (128.98 ± 3.19) was significantly later than that of *A. lobatum* (126.67 ± 4.35) by 2 days (*p* < .01), and they were significantly earlier than that of *A. erubescens* (139.43 ± 4.68) by 18 and 16 days, respectively (both *p* < .001, Figure S2). Flowering phenology overlap of the three *Arisaema* species varied among different species pairs. Flowering phenology of *A. bockii* (41 days) overlapped substantially with that of *A. lobatum* (40 days) for 40 days, and the peaks were close (Figure 2). Flowering phenology of *A. erubescens* (30 days) partially overlapped that of the former two species for 23 days, and the peaks differed (Figure 2).

### 3.2 Pollinator isolation

**FIGURE 3:**
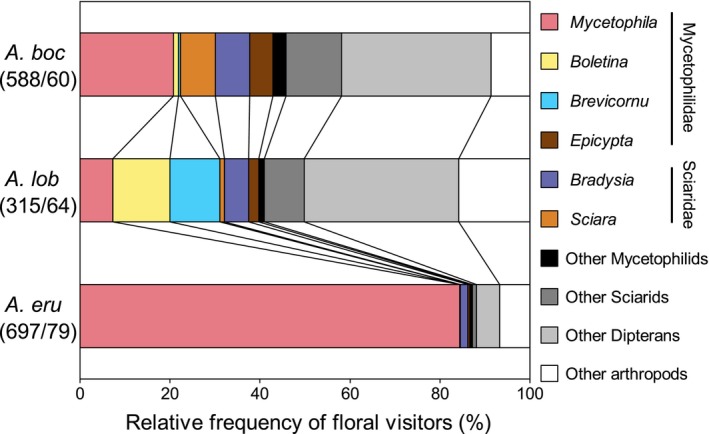
Floral visitor assemblages of the three *Arisaema* species in the Badagongshan Reserve. “Other arthropods” includes arthropods that we were unable to identify. Species abbreviations as in Figure 2 legend.

**FIGURE 4:**
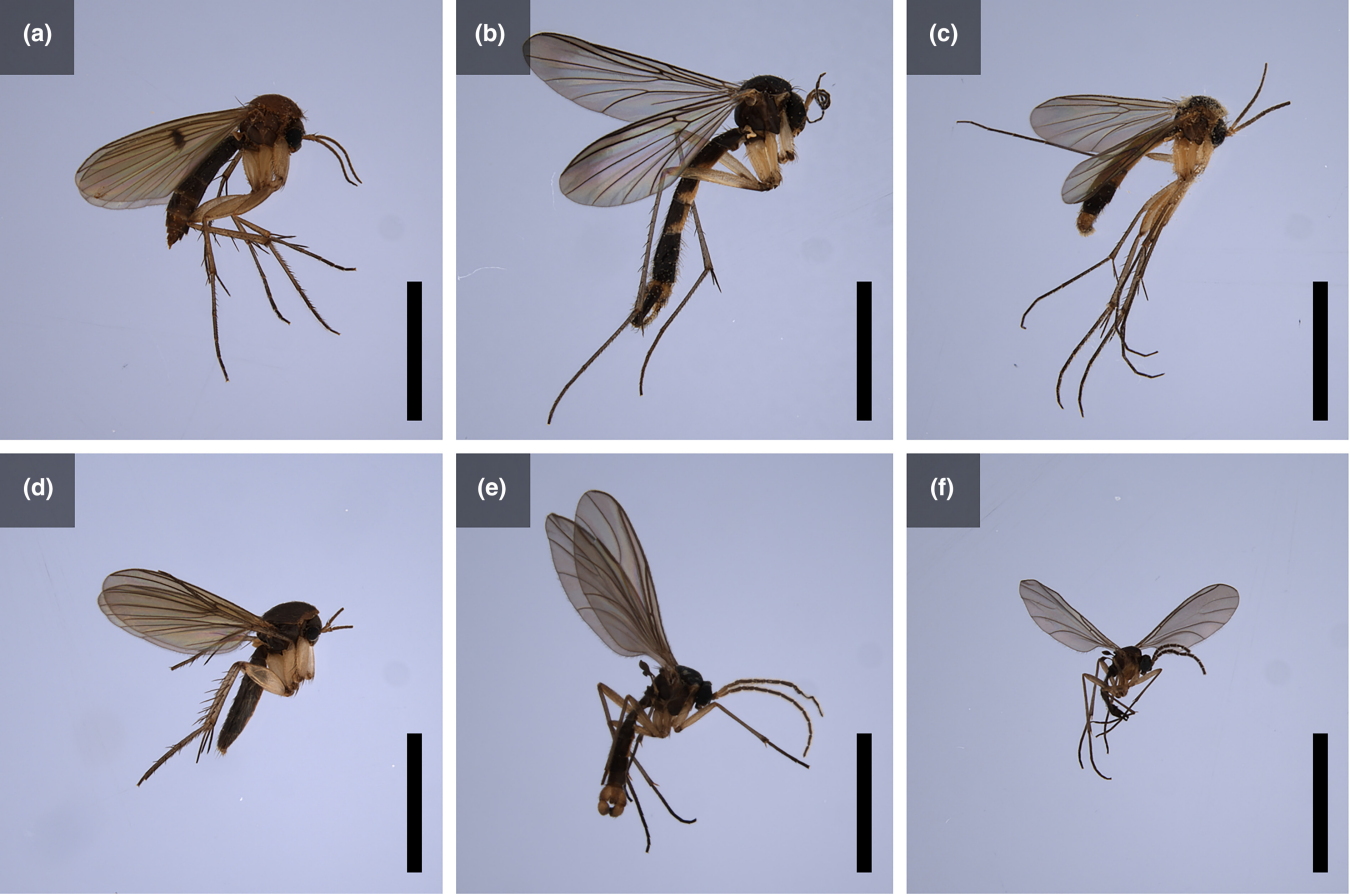
Main fungus gnats visiting the inflorescences of the three *Arisaema* species in the Badagongshan Reserve. (a) *Mycetophila* sp.1. (b) *Boletina* sp.1. (c) *Brevicornu* sp.1. (d) *Epicypta* sp.1. (e) *Sciara* sp.1 (f) *Bradysia* sp.1. Bar = 5 mm.

Floral visitor assemblage differed significantly among the three *Arisaema* species (*R* ^2^ = .20, *p* < .001). All pairs of the three *Arisaema* species were significantly different in the floral visitor assemblage (all *p* < .001). Although for all three *Arisaema* species the most frequent insect visitors were Mycetophilidae and Sciaridae fungus gnats (49.84%–88.09%), the assemblage of fungus gnats differed somewhat among the three *Arisaema* species. Both *A. bockii* and *A. lobatum* attracted various genera of fungus gnats. Some genera of fungus gnats were attracted almost exclusively to *A. bockii* (*Epicypta* spp. and *Sciara* spp.) or *A. lobatum* (*Boletina* spp. and *Brevicornu* spp.), while others were attracted to both *Arisaema* species (*Mycetophila* spp. and *Bradysia* spp.). On the contrary, *A. erubescens* attracted almost exclusively *Mycetophila* spp., which were also attracted to the other two *Arisaema* species (Figure 3). By observing the morphological characteristics of fungus gnats, we found that each genus was dominated by one species (Figure 4), and that if a genus visited the inflorescences of more than one *Arisaema* species, the main species was the same.

### 3.3 Hybrid fruit and seed formation

**FIGURE 5:**
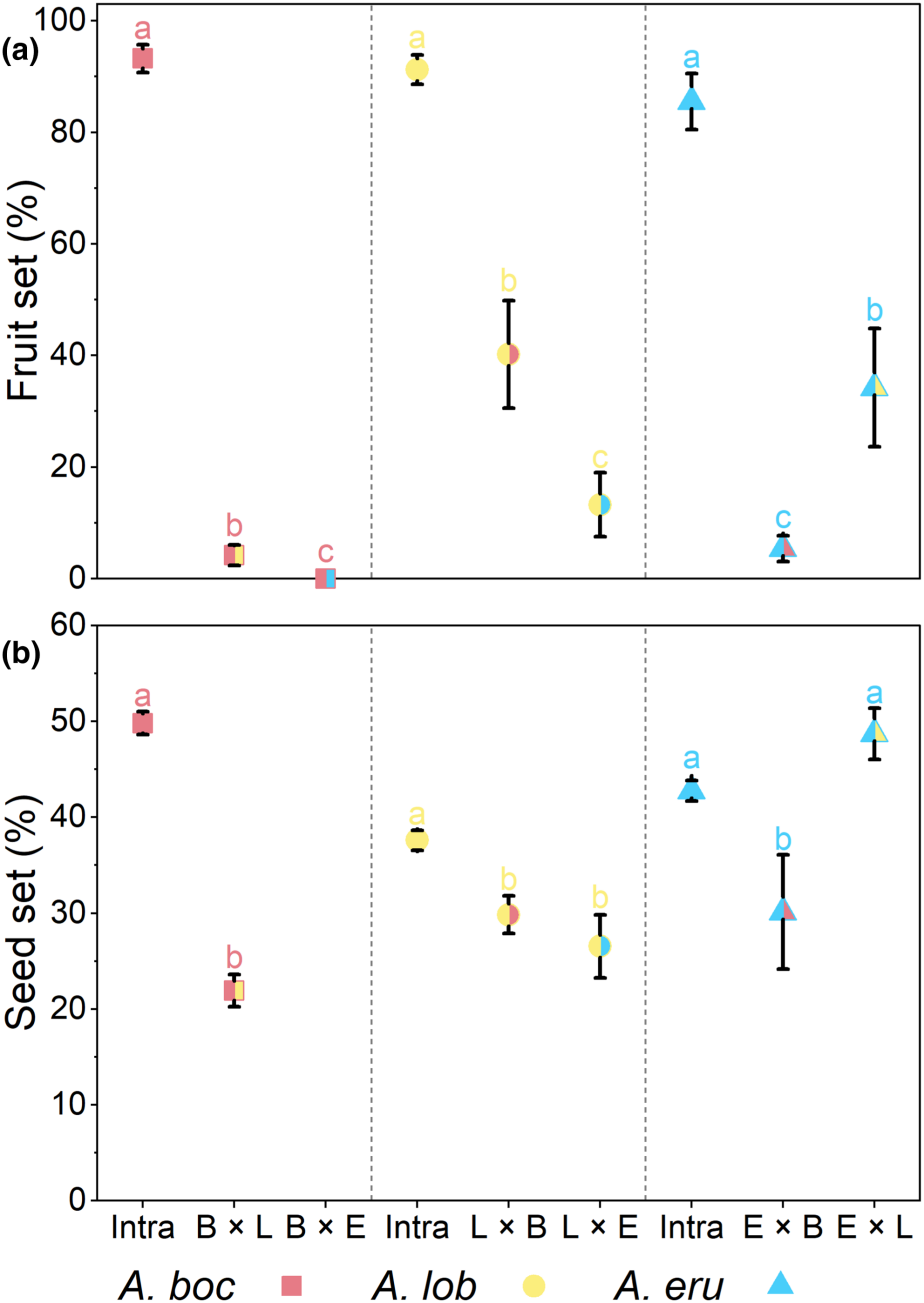
Mean fruit set (a) and seed set (b) (±SEM) obtained from intra‐ and interspecific crosses between the three *Arisaema* species in the Badagongshan Reserve. Letters indicate species and shapes indicate maternal species for each cross: B/square = *A. bockii*, L/circle = *A. lobatum*, E/triangle = *A. erubescens*. Letters over symbols indicate significant differences between treatments within each maternal species. Species abbreviations as in Figure 2 legend.

Fruit sets ranged between 85.55% and 93.26% for intraspecific crosses and between 0% and 40.23% for interspecific crosses. When *A. bockii* was the pollen recipient, fruit sets in interspecific crosses with *A. lobatum* (4.23%, *n* = 218) and *A. erubescens* (0, *n* = 182) were significantly lower than fruit set in intraspecific cross (93.26%, *n* = 434, both *p* < .05). When *A. lobatum* was the pollen recipient, fruit sets in interspecific crosses with *A. bockii* (40.23%, *n* = 204) and *A. erubescens* (13.27%, *n* = 201) were significantly lower than fruit set in intraspecific cross (91.26%, *n* = 415, both *p* < .01). When *A. erubescens* was the pollen recipient, fruit sets in interspecific crosses with *A. bockii* (5.41%, *n* = 206) and *A. lobatum* (34.22%, *n* = 267) were significantly lower than fruit set in intraspecific cross (85.55%, *n* = 514, both *p* < .01, Figure 5).

Seed sets ranged between 37.61% and 49.82% for intraspecific crosses and between 21.93% and 48.72% for interspecific crosses. When *A. bockii* was the pollen recipient, seed set in interspecific cross with *A. lobatum* (20.93%, *n* = 10) was significantly lower than seed set in intraspecific cross (49.82%, *n* = 405, *χ* ^2^ = 18.27, *p* < .001). When *A. lobatum* was the pollen recipient, seed sets in interspecific crosses with *A. bockii* (29.83%, *n* = 88) and *A. erubescens* (26.54%, *n* = 26) were significantly lower than seed set in intraspecific cross (37.61%, *n* = 377, both *p* < .01). When *A. erubescens* was the pollen recipient, seed set in interspecific cross with *A. bockii* (30.13%, *n* = 13) was significantly lower than seed set in intraspecific cross (42.76%, *n* = 436, *χ* ^2^ = 6.18, *p* < .05), and seed set in interspecific cross with *A. lobatum* (48.72%, *n* = 100) was higher than seed set in intraspecific cross with no significant differences (*χ* ^2^ = 3.80, *p* = .051, Figure 5).

### 3.4 Strength of pre‐ and post‐pollination barriers and its contribution to total isolation

**FIGURE 6:**
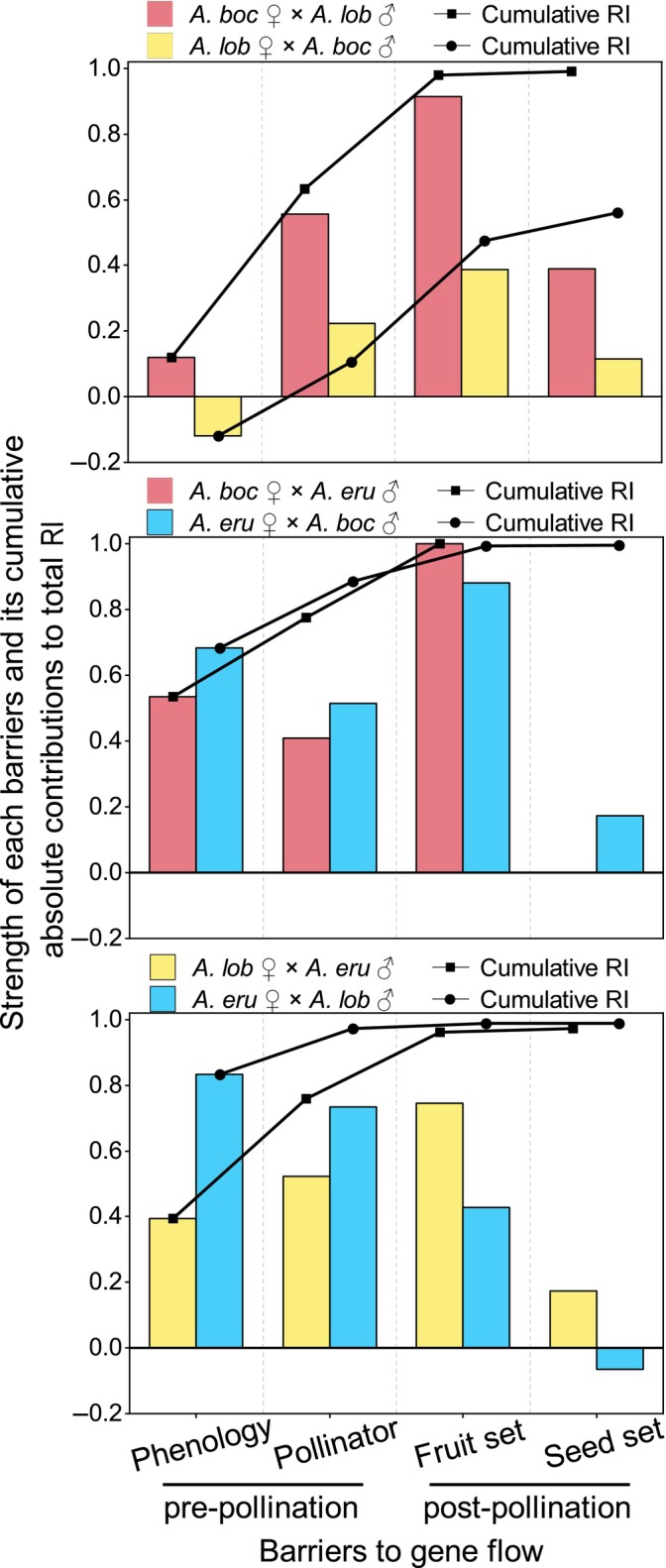
Strength of multiple pre‐ and post‐pollination barriers among the three *Arisaema* species and their absolute contribution to total isolation. The bar graphs represent the strength of individual barrier. The line graph represents the cumulative contribution to reproductive isolation of a mechanism after accounting for each of the investigated previous mechanisms. Species abbreviations as in Figure 2 legend.

The strength of phenological isolation (mean ± SD, 0.41 ± 0.36) varied from −0.12 to 0.83 among the three *Arisaema* species. Reproductive isolation was weak between *A. bockii* and *A. lobatum* (0.12 and −0.12) but strong in the other two species pairs (0.39–0.83). The absolute contribution of phenological isolation was equal to its strength, since we assumed that it occurred before other quantified isolation barriers (Figure 6).

The strength of pollinator isolation (0.49 ± 0.17) was relatively strong in all pairs of the three *Arisaema* species, ranging from 0.22 to 0.73. The absolute contribution of pollinator isolation (0.28 ± 0.14) varied from 0.14 to 0.51 (Figure 6).

The strength of reproductive barrier to hybrid fruit formation was strong (mean ± SD 0.73 ± 0.26), ranging from 0.39 to 1. The absolute contribution of isolation barrier to hybrid fruit formation (0.21 ± 0.14) varied from 0.02 to 0.37 (Figure 6).

The strength of reproductive barrier to hybrid seed formation (mean ± SD 0.16 ± 0.16) varied from −0.07 to 0.39. The absolute contribution of isolation barrier to hybrid seed formation (0.02 ± 0.04) varied from −0.002 to 0.08 (Figure 6).

The cumulative absolute contribution to total RI varied from 0.11 to 0.97 for pre‐pollination barriers and from 0.01 to 0.45 for post‐pollination barriers. The contribution of pre‐pollination isolation (0.69 ± 0.31) to total isolation was significantly higher than that of post‐pollination isolation barriers (0.23 ± 0.16, *p* < .05, Figure 6).

The strength of total isolation (mean ± SD, 0.99 ± 0.01) exceeded 0.97 in most species pairs among the three *Arisaema* species, except for species pair lob♀boc♂ (RI_total_ = 0.56, Figure 6).

## 4 DISCUSSION

### 4.1 Pre‐ and post‐pollination mechanisms of reproductive isolation

Difference in flowering time hinders temporally interspecific pollen transfer when close relatives encounter (Liu et al., 2020; Xu et al., 2020). In this study, the contribution of flowering phenology to reproductive isolation varied in different species pairs. Substantially overlapping flowering phenology led to a weak phenological isolation between *A. bockii* and *A. lobatum*, but staggered flowering phenology led to an effective phenological isolation between the late‐flowering *A. erubescens* and the former two. Overlapping flowering time has also been reported in other sympatric *Arisaema* species pairs (Matsumoto et al., 2019, 2021; Murata & Ohno, 1989). This is not only due to the fact that closely related species have similar flowering times (Du et al., 2015), but may also be related to their pseudoannual life history. This life cycle restricts the time available for growth and reproduction due to the annual death and regrowth of the above‐ground stems, as has been described for other geophytic plant species (Dafni et al., 1981). In fact, 63 of the 70 *Arisaema* species recorded in China bloom between April and July (Li et al., 2010). On the contrary, each of the three species in this study had a long flowering period of more than a month. Long flowering periods need to be maintained for *Arisaema* species living at high altitudes with highly variable weather conditions to ensure successful pollination (Barriault et al., 2009), which makes it easy for flowering periods to overlap between different species in this taxon. In some other taxa (e.g., *Gentiana* and *Primula*); however, individual species remain isolated for brief flowering periods (Rawat, 2012).

Pollinator isolation is divided into mechanical isolation due to the structural contrivances of the flower and behavioral isolation due to the constancy of the pollinating animals to one kind of flower (Grant, 1949). Mechanical isolation is a crucial pre‐pollination isolation in some taxa, such as *Pedicularis* (Huang & Shi, 2013). This is not the case in *Arisaema* species with similar trap flower structures, as in the three species studied. Behavioral isolation has been identified as an important contributor to reproductive isolation in *Arisaema* due to selective fungus gnat visitation. There was strong pollinator isolation among some other *Arisaema* species due to visits by the almost entirely different fungus gnats (Matsumoto et al., 2021; Suetsugu et al., 2021). However, since some genera of fungus gnats in this study were attracted to two or more *Arisaema* species at the same time, the interspecific pollinator isolation remained incomplete. It is believed that *Arisaema* species attract fungus gnats that use the fungus for larval development through fungal mimicry (Kakishima et al., 2019; Vogel & Martens, 2000). Flower visitors collected in his study include *Mycetophila* spp. and *Boletina* spp., both of which are fungivores (Jakovlev, 2012). This result supports the fungal mimicry hypothesis. However, a small number of fungus gnat species are confined to specific genera and species of fungal hosts (Jakovlev, 2012). This may be responsible for the sharing of pollinators among *Arisaema* species. In the future, Chemical studies of the key pollinator‐attracting compounds derived from *Arisaema* inflorescences will provide insights into the mechanism that underlies pollinator isolation.

Intrinsic isolation mechanisms may reduce hybrid fruit and seed formation when interspecific pollen transfer is present (Baack et al., 2015; Christie & Strauss, 2019), as reported for three sympatric *Pedicularis* (Liang et al., 2018). However, they have been identified as weak or absent among *Arisaema* species due to natural hybridization and high hybrid viability reported in some studies (Hayakawa et al., 2011, 2013; Kobayashi et al., 2005; Lee et al., 2011; Maki & Murata, 2001; Murata et al., 2018; Murata & Ohno, 1989; Sanders & Burk, 1992). In this study, the fruit set and seed set of interspecific crosses were significantly lower than those of intraspecific crosses in the artificial crosses experiment, suggesting that the intrinsic post‐pollination barriers to hybrid fruit and seed formation may effectively reduce the production of hybrids among the three *Arisaema* species. The examination of post‐pollination, pre‐zygotic mechanisms was not conducted in this study. Future studies could examine whether there are potential barriers to pollen tube formation and growth. The evolution of intrinsic barriers may be related to the accumulation of genetic incompatibilities and may also be affected by the costs incurred by hybridization. When hybridization is costly, selection should act more strongly against maladaptive gene flow in the direction of the cross that suffers more (Hoskin et al., 2005). Our data indicate that pre‐pollination isolation was not complete, and intrinsic barriers effectively reduce the formation of hybrid fruits. We speculate that complete pre‐pollination isolation may have been difficult to evolve among these species due to similar niches, life patterns, and pollination strategies and that early post‐pollination barriers may have been used as an alternative to avoid more costs incurred by hybridization. Future studies comparing the strength of post‐pollination isolation among these species in sympatric and allopatric distributions could shed light on whether the cost of hybridization drives the evolution of early post‐pollination barriers.

### 4.2 Contribution of pre‐ and post‐pollination isolation to total isolation

Pre‐pollination barriers associated with adaptive ecological divergence were important, while intrinsic post‐pollination barriers associated with the accumulation of genetic incompatibilities contributed little to total isolation in many case studies of reproductive isolation in plants (Christie et al., 2022; Lowry et al., 2008). In this study, the early‐acting pre‐pollination barriers contributed more to reproductive isolation than the post‐pollination barriers in most species pairs among the three *Arisaema* species, although the reproductive barrier to the fruit formation was strong in these species pairs. These results are consistent with other case studies of reproductive isolation in plants (Christie & Strauss, 2019; Karrenberg et al., 2019), and support the idea that ecological divergence as primary drivers of angiosperm divergence and/or the maintenance of contemporary species boundaries (Christie et al., 2022; Lowry et al., 2008). However, ecological divergence does not always contribute more to the maintenance of species integrity. For species pairs *lob*♀*boc*♂, pre‐pollination barriers were weak and early post‐pollination barriers at the fruit and seed formation stage were important contributors to reproductive isolation. Despite incomplete cumulative isolation of species pairs *lob*♀*boc*♂ (RI_total_ = 0.56) in this study, we found only a few of the morphologically intermediate putative hybrids. We suspect that intrinsic and extrinsic late post‐pollination barriers such as hybrid sterility may play a role in maintaining their species boundaries. The difference in chromosome numbers between *A. bockii* (2n = 26) and *A. lobatum* (2n = 28) may reduce the fertility of hybrids, as has been reported for *A. limbatum* (2n = 26) and *A. ringens* (2n = 28) (Kobayashi et al., 2005).

Effective pre‐pollination barriers are expected to prevent interspecific gene flow among sympatric *Arisaema* species (Matsumoto et al., 2019, 2021; Murata et al., 2018; Suetsugu et al., 2021). For example, in 18 of the 20 species pairs among five *Arisaema* species examined, the strength of total pre‐pollination isolation exceeded 0.96 (Matsumoto et al., 2021). In contrast, post‐pollination barriers are considered to be weak or absent among *Arisaema* species (Matsumoto et al., 2019; Murata et al., 2018). However, in this study, pre‐pollination barriers were not able to completely prevent interspecific gene flow among the three *Arisaema* species, although phenological isolation and pollinator isolation effectively reduced interspecific pollen transfer. Strong post‐pollination isolation also played an important role in preventing interspecific gene flow among the three *Arisaema* species, particularly in the reproductive isolation between *A. bockii* and *A. lobatum*. Incomplete pre‐pollination isolation may be due to the difficulty of ecological differentiation among these species due to similar niches, life patterns, and pollination strategies. Strong post‐pollination barriers serve as a safeguard against interspecific gene flow to maintain species boundaries. Both pre‐ and post‐pollination barriers are required to effectively prevent hybridization and maintain the integrity of the three sympatric *Arisaema* species.

## 5 CONCLUSION

In this study, we quantified multiple pre‐ and post‐pollination barriers and their absolute contribution to total isolation among three sympatric *Arisaema* species in the Badagongshan Reserve. Although phenological isolation and pollinator isolation significantly reduced frequencies of interspecific pollen transfer, pre‐pollination reproductive barriers may be insufficient to prevent interspecific hybridization among the three *Arisaema* species. Our study is the first to show the effective reproductive barriers to hybrid fruit and seed formation between *Arisaema* species. Our study confirms that reproductive isolation among three sympatric *Arisaema* species is a result of a combination of pre‐ and post‐pollination barriers. Future studies of reproductive isolation in *Arisaema* should include multiple pre‐ and post‐pollination reproductive barriers of a broader range of species to determine whether post‐pollination reproductive barriers play an important role in the maintenance of contemporary species boundaries in this large genus.

## AUTHOR CONTRIBUTIONS

**Luo Zeng:** Data curation (equal); investigation (equal); writing – original draft (equal). **Wei‐Jie Shu:** Investigation (supporting). **Hua He:** Investigation (supporting). **Tao Li:** Investigation (supporting). **Xiao‐Chen Yang:** Conceptualization (equal); supervision (equal); writing – review and editing (equal). **Li Li:** Funding acquisition (lead); supervision (equal); validation (lead).

## CONFLICT OF INTEREST STATEMENT

The authors declare that there are no potential conflicts of interest regarding this research.

## Supporting information


Figure S1

Figure S2

Table S1

Table S2

Table S3

## Data Availability

The data that supports the findings of this study are available in the supplementary material of this article.
