# Supplementary figures and images for: Post‐pollination barriers contribute to coexistence of partially pollinator‐sharing Arisaema species (Araceae)
Source: Ecol Evol. 2023 Nov 2;13(11):e10696. doi: 10.1002/ece3.10696 (PMC10620566; doi:10.1002/ece3.10696)

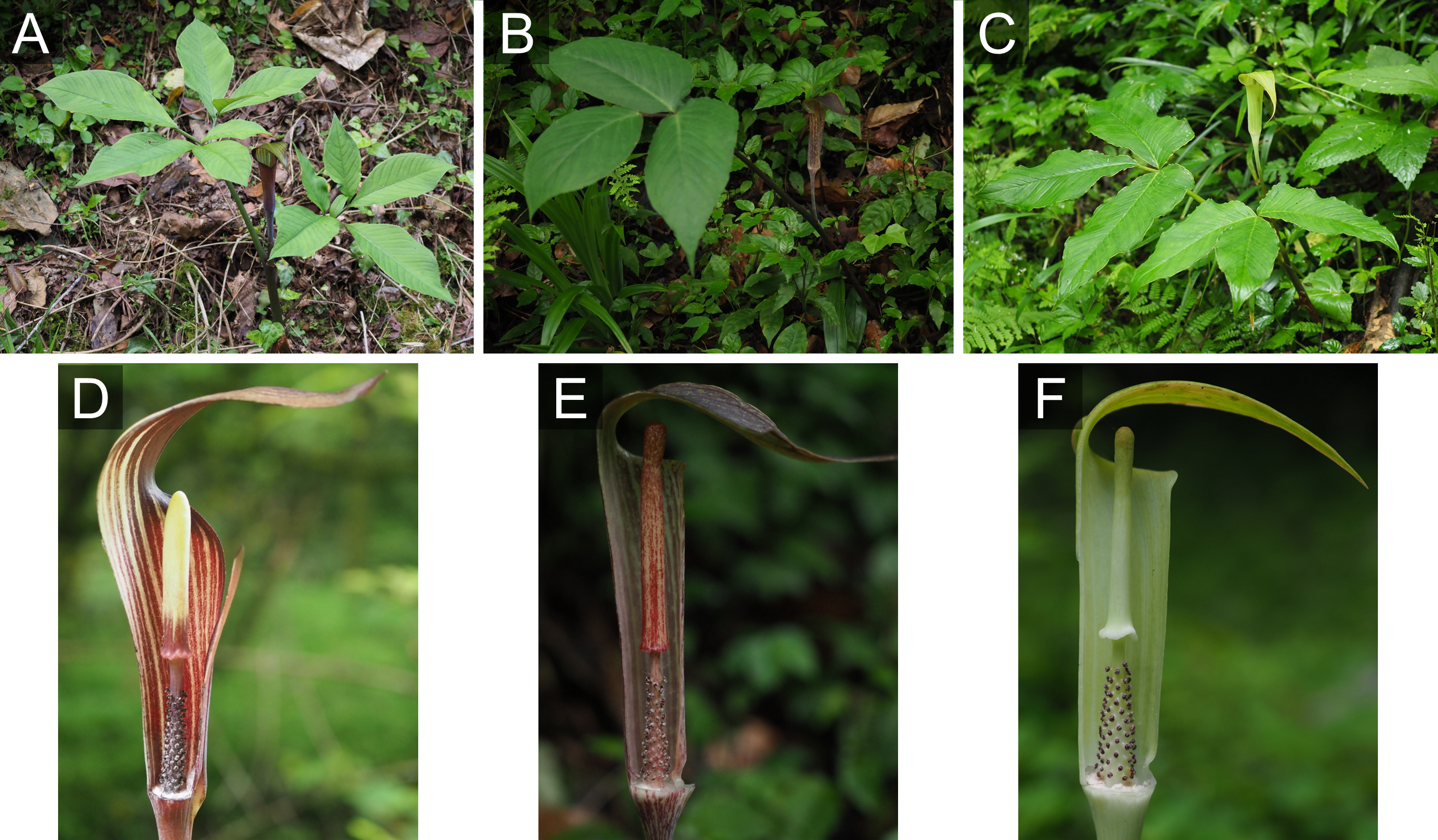

Supplement: Supplementary file 1 — Figure S1 [file ECE3-13-e10696-s003.tif]

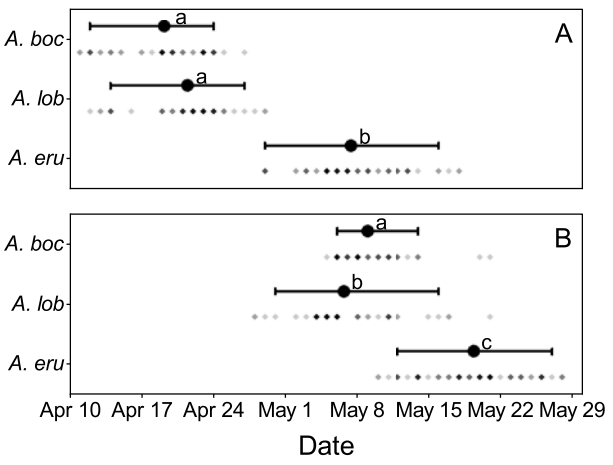

Supplement: Supplementary file 2 — Figure S2 [file ECE3-13-e10696-s001.pdf]
